# Isolation of Vaginal Lactobacilli and Characterization of Anti-*Candida* Activity

**DOI:** 10.1371/journal.pone.0131220

**Published:** 2015-06-22

**Authors:** Carola Parolin, Antonella Marangoni, Luca Laghi, Claudio Foschi, Rogers Alberto Ñahui Palomino, Natalia Calonghi, Roberto Cevenini, Beatrice Vitali

**Affiliations:** 1 Department of Pharmacy and Biotechnology, University of Bologna, Bologna, Italy; 2 Microbiology, DIMES, University of Bologna, Bologna, Italy; 3 Centre of Foodomics, Department of Agro-Food Science and Technology, University of Bologna, Bologna, Italy; Louisiana State University, UNITED STATES

## Abstract

Healthy vaginal microbiota is dominated by *Lactobacillus* spp., which form a critical line of defence against pathogens, including *Candida* spp. The present study aims to identify vaginal lactobacilli exerting *in vitro* activity against *Candida* spp. and to characterize their antifungal mechanisms of action. *Lactobacillus* strains were isolated from vaginal swabs of healthy premenopausal women. The isolates were taxonomically identified to species level (*L*. *crispatus* B1-BC8, *L*. *gasseri* BC9-BC14 and *L*. *vaginalis* BC15-BC17) by sequencing the 16S rRNA genes. All strains produced hydrogen peroxide and lactate. Fungistatic and fungicidal activities against *C*. *albicans*, *C*. *glabrata*, *C*. *krusei*, *C*. *tropicalis*, *C*. *parapsilosis* and *C*. *lusitaniae* were evaluated by broth micro-dilution method. The broadest spectrum of activity was observed for *L*. *crispatus* BC1, BC4, BC5 and *L*. *vaginalis* BC15, demonstrating fungicidal activity against all isolates of *C*. *albicans* and *C*. *lusitaniae*. Metabolic profiles of lactobacilli supernatants were studied by ^1^H-NMR analysis. Metabolome was found to be correlated with both taxonomy and activity score. Exclusion, competition and displacement experiments were carried out to investigate the interference exerted by lactobacilli toward the yeast adhesion to HeLa cells. Most *Lactobacillus* strains significantly reduced *C*. *albicans* adhesion through all mechanisms. In particular, *L*. *crispatus* BC2, *L*. *gasseri* BC10 and *L*. *gasseri* BC11 appeared to be the most active strains in reducing pathogen adhesion, as their effects were mediated by both cells and supernatants. Inhibition of histone deacetylases was hypothesised to support the antifungal activity of vaginal lactobacilli. Our results are prerequisites for the development of new therapeutic agents based on probiotics for prophylaxis and adjuvant therapy of *Candida* infection.

## Introduction

The homeostasis of the vaginal ecosystem results from complex interactions and synergies among the host and different microorganisms that colonize the vaginal mucosa [1, 2]. Healthy vaginal microbiota is generally dominated by *Lactobacillus* spp. [3, 4]. These bacteria form a critical line of defence against potential pathogens by producing antimicrobial compounds, or through competition for adherence to the vaginal epithelium [5–8]. For the positive effects of lactobacilli on the health of female genital tract there is an increasing interest for their use in probiotic formulations for the prophylaxis and therapy of several vaginal disturbances [9–11].

Vulvovaginal candidiasis (VVC) is a common infection compromising the quality of life of many women. *Candida albicans* is the most frequent etiologic agent [12]. Information on VVC incidence is incomplete, since the disease is not reportable and data collection is hampered by inaccuracies of diagnosis and the use of non-representative study populations. Thus, the extent to which VVC is a source of population-level morbidity remains uncertain [13]. Although the pathogenesis of VVC remains a controversial issue, it seems that, when the balance of the vaginal microbiota is disrupted, the overgrowth of *Candida* spp. is facilitated. Individual susceptibility, pregnancy, antibiotic therapy, use of contraceptives and spermicide, frequent sexual intercourse, diabetes and immunosuppression are factors that increase the risk for development of VVC [14–16]. Infections by *C*. *albicans* are commonly treated with azole antifungal drugs. Since azoles are fungistatic for *C*. *albicans*, cells repetitively exposed to these antifungals adapt to the drug pressure and become azole resistant [17]. The high incidence of VVC together with the growing problem of drug resistance highlight the need for the development of new effective agents for the prevention and therapy of this gynecological infection [18].

Establishment of a healthy vaginal microbiota using probiotic *Lactobacillus* strains might be a supportive and preventive measure against VVC [19]. Lactobacilli are supposed to protect from *Candida* infection but the mechanisms underlying antifungal activity are still not clearly understood. Although lactobacilli are quite common even in the vaginal epithelium of women with VVC, the composition of *Lactobacillus* species/strain is different compared to healthy women. In particular, the development of VVC has been associated with the lack of H_2_O_2_-producing *Lactobacillus* species [20].

The capacity of lactobacilli to adhere and compete for the adhesion sites on vaginal mucosa can be involved in the inhibition of *Candida* colonization. The blockage of *Candida* adherence may be by exclusion, competition for receptors sites and displacement of adhered yeast cells [21–24]. Several studies have shown that the inhibition of histone deacetylases (HDACs) can affect morphogenesis, attenuate the virulence and reduce the adhesion of *Candida* spp. to host mucosa [25–27], in addition to enhancing the antifungal activity of azole drugs [28]. Butyrate and lactate are known HDAC inhibitors [29, 30]. Since these metabolites are produced by several human commensal bacteria, the anti-*Candida* activity of the vaginal lactobacilli could be mediated by the inhibition of HDACs.

The present study aims to isolate vaginal lactobacilli from healthy women and to evaluate their ability to counteract the infection by *Candida* spp. In particular, we investigated possible antifungal mechanisms of action, i.e, the production of antimicrobial compounds, the interference with adhesion process and the inhibition of HDACs. We used a metabolomic approach based on ^1^H-NMR [31] to find correlations between metabolism of lactobacilli and anti-*Candida* activity. A major potential application of this study concerns the identification of active *Lactobacillus* strains to propose as probiotics for prophylaxis and/or adjuvant therapy of VVC.

## Materials and Methods

### Isolation of vaginal lactobacilli from healthy women and taxonomic characterization

Fifteen pre-menopausal Caucasian women (aged 18–45 years old), who have no symptoms of vaginal or urinary tract infection, were recruited for the present study. The women were non-menstruating and not receiving oral or local antimicrobial therapy within the previous 2 weeks. All volunteers provided a written informed consent in accordance with the Ethics Committee of the University of Bologna (52/2014/U/Tess) and the institutional review board approved the study. Mid-vaginal secretions were self-collected by women with E-swabs (Copan, Brescia, Italy) and immediately processed for lactobacilli isolation. The specimens were coded to assure full anonymousness.

*Lactobacillus* clones were isolated onto de Man, Rogosa and Sharpe (MRS) and Brain-Heart Infusion (BHI) agar plates (Difco, Detroit, MI). Both MRS and BHI agar plates were supplemented with 0.05% L-cysteine. Plates were incubated anaerobically for 24 h at 37°C in anaerobic jars supplemented with Anaerocult C (Merck, Milan, Italy). Colonies with different morphologies yielding variable rods by microscope observation were selected for glycerol stock preparation. To prepare lactobacilli fractions, 18-h MRS/BHI cultures (OD_600_ = 0.5) were centrifuged at 5,000 X *g* for 10 min at 4°C. Supernatants were filtered through a 0.2 μm membrane filter to obtain cell free supernatants (CFS). Cell pellets (CP) were washed in sterile saline.

Genomic DNA was extracted from lactobacilli CP using DNeasy Blood & Tissue Kit (Qiagen, Hilden, Germany) following the protocol “Pretreatment for Gram-positive bacteria”. The extracted DNA was amplified with *Lactobacillus* genus-specific primers Lac1 and Lac2 [32]. The positive isolates were taxonomically characterized to the species level by sequencing the 16S ribosomal RNA (rRNA) gene. Briefly, the complete 16S rRNA gene (1.5 kb) was amplified with the universal primers 27F and 1492R [33] and sequenced. The obtained sequences were compared with the sequences available in the Ribosomal Database Project (RDP, http://rdp.cme.msu.edu/) [34] in order to identify the *Lactobacillus* species.

### Determination of hydrogen peroxide

*Lactobacillus* strains were tested for their ability to produce H_2_O_2_ as described by Pendharkar *et al*. [35] with slight modifications. Isolates were cultured onto MRS agar plate containing 0.25 mg/ml 3,3’, 5,5’-tetramethylbenzidine and 0.01 mg/ml of horseradish peroxidase in anaerobic condition for 72 h. Plates were exposed to air and on the basis of the time required for the blue coloration to appear, isolates were scored as low [score 1(>20 min)], medium [score 2 (10–20 min)] and high producing strains [score 3 (<10 min)]. Isolates not producing blue coloration were scored as 0.

### ^1^H-NMR analysis

One ml of CFS obtained from lactobacilli was added to 160 μl of a D_2_O solution of 3-(trimethylsilyl)-propionic-2,2,3,3-d4 acid sodium salt (TSP) 6.25 mM set to pH 7.0 by means of a 100 mM phosphate buffer. ^1^H-NMR spectra were recorded at 298 K with an AVANCE III spectrometer (Bruker, Milan, Italy) operating at a frequency of 600.13 MHz. To avoid the presence of broad signals arising from slowly tumbling molecules, a T_2_ filter of 400 echoes, separated by an echo time of 400 μs, was applied. The signals were assigned by comparing their chemical shift and multiplicity with Chenomx software data bank (Chenomx Inc., Canada, ver 8.02). Literature on previous quantitative investigations conducted with the same technique reports a precision error below 2% [36].

### Assessment of fungistatic and fungicidal activities

*Candida* strains used in the present study were part of a broad collection including yeasts isolated from vaginal swabs submitted to the Microbiology Laboratory of Sant’Orsola-Malpighi University Hospital of Bologna for routine diagnostic procedures. In particular, 4 isolates of *C*. *albicans*, and 1 isolate of each from the following species were used: *C*. *glabrata*, *C*. *krusei*, *C*. *tropicalis*, *C*. *parapsilosis* and *C*. *lusitaniae*. All the clinical isolates were coded to assure full anonymousness (S1 Table). *Candida* strains were grown aerobically in Sabouraud dextrose (SD) medium (Oxoid, Basingstoke, Hampshire, UK) at 35°C.

The fungistatic activity of *Lactobacillus* CFS was determined by broth microdilution in accordance with the EUCAST guidelines [37] with slight modifications. Briefly, *Candida* suspensions were prepared in sterile water from 24-h cultures on SD agar and the turbidity was adjusted to OD_530_ = 0.5. RPMI 1640 medium buffered to pH 7.0 with 0.165 M morpholinepropanesulfonic acid buffer and 2% glucose was added to give a yeast suspension of 1–5 × 10^5^ CFU/ml. Each well of a flat-bottom microdilution tray was inoculated with 100 μl of yeast suspension, and subsequently filled with 100 μl of *Lactobacillus* CFS. A growth control well contained 100 μl of sterile MRS medium and 100 μl of the same *Candida* suspension. The microdilution trays were incubated at 35°C and growth was observed after 24 and 48 h. The results were read considering a prominent decrease in turbidity (at least 50% reduction in growth) relative to the control by measuring the absorbance at 450 nm with Multiskan FC Microplate Photometer (Thermo Fisher Scientific Inc.,Waltham, USA). To determine a fungicidal effect of *Lactobacillus* CFS, 20 μl of samples from wells exhibiting less than 50% of growth were spotted onto SD agar plates and incubated at 35°C for 24/48 h. Fungicidal activity was defined as a ≥ 3 log_10_ reduction from the starting inoculum [38]. Following the same methods, fungistatic/fungicidal activities were tested for all the compounds identified by ^1^H-NMR as differently expressed among the lactobacilli.

### Adhesion assays

HeLa cells were grown to confluent monolayers in 5% CO_2_ at 37°C, inside a Forma Series II 3110 Water-Jacketed CO_2_ Incubator (Thermo Fisher Scientific Inc.,Waltham, USA), in Dulbecco’s minimal essential medium (DMEM) (EuroClone, Pero, Italy), supplemented with 10% foetal bovine serum, 1% L-glutamine, 100 IU/ml penicillin G and 100 μg/ml streptomycin.

Capability of each *Lactobacillus* strain to adhere to HeLa cells was evaluated in individual tubes containing sterile coverslips as previously reported [24, 39] with slight modifications. One millilitre of HeLa cell suspension, at a concentration of 5 × 10^4^ cells/ml, was seeded onto each glass coverslip and incubated in 5% CO_2_ atmosphere at 37°C. After 48 h, the cells, grown to approximately 70% confluence, were washed twice with PBS and treated with 100 μl of lactobacilli suspension (5 × 10^8^ bacteria/ml). The tubes were then incubated for 1 h at 37°C in 5% CO_2_. Cell monolayers were washed several times in PBS, fixed with May-Grünwald and stained with Giemsa. Results were read at light-microscopy (1000×) and HeLa cells were scored for the presence and number of lactobacilli attached. Each adherence assay was conducted in duplicate and 200 randomly chosen cells were evaluated for lactobacilli adhesion.

To study the interference of vaginal lactobacilli with the adherence of *Candida* to HeLa cells, *C*. *albicans* 1 was chosen as model strain. Yeast culture in BHI broth was incubated at 30°C for 18 h under constant shaking, in order to obtain blastospores at late exponential growth phase [23]. Yeast cells were collected by centrifugation, washed three times and finally suspended in saline solution to the working dilution of 5 × 10^8^ yeasts/ml. Three types of assays were performed to study the capacity of *Lactobacillus* fractions (CP and CFS) to interfere with the adherence of *C*. *albicans* to HeLa cells: exclusion, competition and displacement [21]. In the adhesion assays, CP and CFS fractions corresponding to 5 × 10^7^ *Lactobacillus* cells were incubated with 5 × 10^7^ *Candida* cells following the timelines described, as follows. In the exclusion assay, lactobacilli fractions were incubated for 1 h at 37°C on HeLa cells. Afterwards, *Candida* cells were added and further incubated for 1 h. In the competition assay, lactobacilli fractions and *Candida* were inoculated simultaneously onto HeLa cells and incubated for 1 h at 37°C. In the displacement assay, *Candida* cells were inoculated onto HeLa cells for 1 h at 37°C. Successively, lactobacilli fractions were added and further incubated for 1 h. Yeast adhesion to HeLa cells was assessed by microscopy (400×) after Giemsa staining by counting the number of *Candida* cells attached to 200 randomly chosen cells. Results were expressed as the percentage of *C*. *albicans* adherent to each HeLa cell and compared with adhesion without lactobacilli fractions (control value = 100%). Interference experiments were conducted three times with at least three replicates per group.

### Histone acetylation profile analysis

*Candida albicans* 1 was chosen as model strain to study the histone acetylation profile induced by vaginal lactobacilli. Log-phase *Candida* cells were inoculated at an OD_600_ of 0.5 in lactobacilli CFS or MRS broth (negative control) and incubated at 30°C for 6 h. Sodium Butyrate (20 mM) was used as positive control, a culture of *Staphylococcus aureus* in MRS was used as a representative Gram-positive organism. Histones were extracted from yeast cultures as described by Knapp *et al*. [40] with slight modifications. A volume equivalent to 20–40 OD units of each culture was collected and subjected to nuclei isolation. Nuclei were washed for 15 minutes on ice in Wash Buffer (10 mM Tris–HCl, pH 8/75 mM NaCl/30 mM Na-Butyrate/0.5% NP-40/1.0 mM PMSF/10 μg/ml each of protease inhibitors). Washes were repeated four times. Histones were acid extracted by incubating nuclei in H_2_SO_4_ 0.4N for 1 h in ice, then precipitated overnight in acetone at -20°C. Equal amount of histones were loaded to a 15% acrylamide gel and separated by SDS-PAGE, then transferred to a nitrocellulose membrane and probed with anti-acetyl Lysine primary antibody (Merck Millipore, Darmstadt, Germany) and peroxidase-conjugated anti-mouse IgG secondary antibody (GE Healthcare, Milan, Italy). Peroxidase activity was detected by Westar XT system (Cyanagen, Bologna, Italy). Digital images and densitometric analysis were performed by using the GS-800 calibrated densitometer (Bio-Rad Laboratories, Milan, Italy). For each strain, histone acetylation profile was analysed in triplicate.

### Statistical analysis

Differences in the metabolome composition were assessed by means of a two-tailed unpaired Wilcoxon test, through the homonym function implemented in R computational software (www.r-project.org). Linear correlations between fungistatic/fungicidal activities and metabolome were assessed by means of ANOVA test. Statistical analyses for the adhesion assays data were performed by using ANOVA test (GraphPad Prism version 5.02 for Windows, GraphPad Software, San Diego California USA, www.graphpad.com). Results were expressed as mean ± Standard Error of the Mean (SEM). Differences were deemed significant for *P* values < 0.05 or highly significant for *P* values < 0.01.

### Nucleotide sequence accession numbers

The nucleotide sequences of the 16S rRNA genes of the *Lactobacillus* strains BC1 to BC17 have been deposited in the DDBJ nucleotide sequence database under accession numbers AB976542 to AB976558.

## Results

### Taxonomy of vaginal lactobacilli and production of antimicrobial compounds

Seventeen *Lactobacillus* isolates were obtained from vaginal swabs of 15 healthy premenopausal women. All isolates were cultured on MRS supplemented with L-cysteine, except *L*. *gasseri* BC14 which was cultured on BHI supplemented with L-cysteine. The *Lactobacillus* isolates were taxonomically identified to species level by sequencing the 16 rRNA gene: 8 isolates belong to *L*. *crispatus* (BC1-BC8), 6 isolates to *L*. *gasseri* (BC9-BC14) and 3 isolates to *L*. *vaginalis* (BC15-BC17) (Table 1).

**Table 1:** Characterization of the vaginal lactobacilli: taxonomy, pH of cultural supernatants and production of antimicrobial compounds.

**Species**	**Strain**	**pH**	**H**_**2**_ **O**_**2**_ **(score)**	**Lactate (mM)**	**Butyrate (mM)**
*L*. *crispatus*	BC1	3.93	3	2.91	3.48 x 10^-1^
*L*. *crispatus*	BC2	4.13	3	6.83	0.00
*L*. *crispatus*	BC3	4.21	1	9.45	0.00
*L*. *crispatus*	BC4	3.87	1	3.32	3.54 x 10^-1^
*L*. *crispatus*	BC5	3.70	2	5.10	1.25 x 10^-1^
*L*. *crispatus*	BC6	4.03	2	7.87	8.33 x 10^-1^
*L*. *crispatus*	BC7	3.91	1	1.42	1.35 x 10^-2^
*L*. *crispatus*	BC8	4.08	1	3.05	4.64 x 10^-1^
*L*. *gasseri*	BC9	3.90	2	4.75	0.00
*L*. *gasseri*	BC10	4.54	3	9.40	0.00
*L*. *gasseri*	BC11	4.20	3	14.6	0.00
*L*. *gasseri*	BC12	4.17	3	9.47	0.00
*L*. *gasseri*	BC13	3.87	1	1.62	1.84 x 10^-2^
*L*. *gasseri*	BC14	4.74	nd*	36.3	1.00 x 10^-2^
*L*. *vaginalis*	BC15	3.95	1	47.4	6.42 x 10^-1^
*L*. *vaginalis*	BC16	4.59	1	24.4	0.00
*L*. *vaginalis*	BC17	4.28	3	23.4	3.27 x 10^-1^

*nd: not determined

As a first step in the characterization of the antimicrobial properties of the vaginal lactobacilli isolated in this study, we evaluated the pH of the cultural supernatants, production of H_2_O_2_, lactate and butyrate (Table 1). The pH of the supernatants was in the range 3.7–4.7, showing the ability of all strains to acidify the medium. *Lactobacillus* isolates were scored for H_2_O_2_ production on a scale of 0 to 3. *Lactobacillus gasseri* BC14 was not tested for H_2_O_2_ production since its incapacity of growing on MRS agar plates and it is important to underline that the hydrogen peroxide test cannot be carried out in BHI plates because tetramethylbenzidine and horseradish peroxidase precipitate in the form of crystals in this medium. Hydrogen peroxide was produced by the totality of the strains. The levels of H_2_O_2_ production did not seem related to a particular species. The strongest H_2_O_2_-producers (score 3) were *L*. *crispatus* BC1, BC2, *L*. *gasseri* BC10, BC11, BC12, and *L*. *vaginalis* BC17. Lactate and butyrate were measured in CFS of lactobacilli cultures by ^1^H-NMR analysis. Lactate was produced by all isolates at concentrations ranging from 1.42 to 47.4 mM. In general, *L*. *vaginalis* species was characterized by good production levels, in particular *L*. *vaginalis* BC15 was the highest producer strain. Instead, *L*. *crispatus* and *L*. *gasseri* species showed variable trends of lactate production. Butyrate was produced by 9 of the 17 lactobacilli at concentrations ranging from 1.00 x 10^-2^ to 8.33 x 10^-1^ mM. Production of butyrate appeared to be negligible in *L*. *gasseri* species and variable in the other two species. The highest levels of this metabolite were found in the supernatants of *L*. *crispatus* BC6 and *L*. *vaginali*s BC15.

### Lactobacilli fungistatic and fungicidal activities

The fungistatic and fungicidal activities of CFS of the vaginal lactobacilli were evaluated against 4 clinical isolates of *C*. *albicans* and 5 clinical isolates referring to species different from *C*. *albicans* (Table 2, S2 Table). In function of the number of *Candida* isolates inhibited by lactobacilli supernatants, the fungistatic and fungicidal activities were scored on a scale of 0 to 4 for *C*. *albicans* and 0 to 5 for non-*C*. *albicans* (Table 3). In general, the strains tested were more active toward *C*. *albicans*. No *Lactobacillus* strains showed activity against *C*. *krusei* and *C*. *parapsilosis*. Even, *L*. *gasseri* BC14 appears to stimulate the growth of *C*. *tropicalis* and *C*. *krusei* probably through production of metabolites that act as growth factors for these two species of *Candida* (S2 Table). The strains that showed the broadest spectrum of anti-*Candida* activity were *L*. *crispatus* BC1, BC4, BC5 and *L*. *vaginalis* BC15, since they had fungicidal activity against all the isolates of *C*. *albicans* and against *C*. *lusitaniae* strain. In addition, *L*. *crispatus* BC1 and *L*. *vaginalis* BC15 exhibited fungistatic activity towards *C*. *tropicalis* and *C*. *glabrata*, showing the best profile of anti-*Candida* activity. *Lactobacillus crispatus* BC4 and BC5 were fungistatic towards only one of *C*. *tropicalis* and *C*. *glabrata* species. A good spectrum of activity was also shown by *L*. *crispatus* BC7 which was fungicidal for all *C*. *albicans* isolates and fungistatic for *C*. *tropicalis* and *C*. *glabrata*. The less active strains were *L*. *gasseri* BC10, BC11, BC14 and *L*. *vaginalis* BC16. *L*. *gasseri* BC10 and BC11 showed no fungistatic/fungicidal activity towards any of *Candida* isolates. *Lactobacillus vaginalis* BC16 exerted a fungistatic activity only towards *C*. *albicans* 1 while *L*. *gasseri* BC14 was fungistatic for *C*. *albicans* 1 and *C*. *glabrata*. Among the remaining lactobacilli, exhibiting an intermediate profile of antifungal activity, the most interesting were *L*. *crispatus* BC3 and BC6 (fungicidal against 3 species of *C*. *albicans* and *C*. *lusitaniae*), and *L*. *crispatus* BC2 (fungicidal against 2 species of *C*. *albicans* and *C*. *lusitaniae*). In summary, the anti-*Candida* activity of lactobacilli CFS was strongly associated with *L*. *crispatus* sp. because all strains of this species were moderately or highly active against the vaginal pathogen. Conversely, poor anti-yeast activity was exhibited by CFS of *L*. *gasseri* spp. Notably, *L*. *vaginalis* spp. showed extremely variable profiles of antifungal activity, comprising one highly active strain, one with an intermediate spectrum, whereas the last one was poor active.

**Table 2:** Fungistatic/fungicidal activity of *Lactobacillus* strains against *Candida* isolates.

*Lactobacillus* strain	*C*. *albicans 1*	*C*. *albicans 2*	*C*. *albicans 3*	*C*. *albicans 4*	*C*. *tropicalis*	*C*. *krusei*	*C*. *parapsilosis*	*C*. *glabrata*	*C*. *lusitaniae*
BC1	+/+	+/+	+/+	+/+	+/-	-/-	-/-	+/-	+/+
BC2	+/-	+/+	+/+	+/-	-/-	-/-	-/-	-/-	+/+
BC3	+/+	+/+	+/-	+/+	-/-	-/-	-/-	-/-	+/+
BC4	+/+	+/+	+/+	+/+	-/-	-/-	-/-	+/-	+/+
BC5	+/+	+/+	+/+	+/+	+/-	-/-	-/-	-/-	+/+
BC6	+/-	+/+	+/+	+/+	+/-	-/-	-/-	+/-	+/+
BC7	+/+	+/+	+/+	+/+	+/-	-/-	-/-	+/-	-/-
BC8	+/-	+/+	+/-	+/-	-/-	-/-	-/-	-/-	+/+
BC9	+/-	+/+	+/-	+/-	-/-	-/-	-/-	-/-	-/-
BC10	-/-	-/-	-/-	-/-	-/-	-/-	-/-	-/-	-/-
BC11	-/-	-/-	-/-	-/-	-/-	-/-	-/-	-/-	-/-
BC12	+/-	-/-	+/-	+/-	-/-	-/-	-/-	-/-	+/+
BC13	+/-	+/-	+/-	+/-	-/-	-/-	-/-	-/-	+/+
BC14	+/-	-/-	-/-	-/-	-/-	-/-	-/-	+/-	-/-
BC15	+/+	+/+	+/+	+/+	+/-	-/-	-/-	+/-	+/+
BC16	+/-	-/-	-/-	-/-	-/-	-/-	-/-	-/-	-/-
BC17	+/-	+/+	-/-	+/-	-/-	-/-	-/-	-/-	+/+

First symbol, fungistatic activity; second symbol, fungicidal activity.

**Table 3:** Fungistatic and fungicidal activity scores of lactobacilli towards *C*. *albicans* and *C*. non-*albicans* isolates.

	**Anti-*Candida* activity score**			
***Lactobacillus* strain**	*C*. *albicans*		*C*. non-*albicans*	
	Fungistatic	Fungicidal	Fungistatic	Fungicidal
BC1	4	4	3	1
BC2	4	2	1	1
BC3	4	3	1	1
BC4	4	4	2	1
BC5	4	4	2	1
BC6	4	3	3	1
BC7	4	4	2	0
BC8	4	1	1	1
BC9	4	1	0	0
BC10	0	0	0	0
BC11	0	0	0	0
BC12	3	0	1	1
BC13	4	0	1	1
BC14	1	0	1	0
BC15	4	4	3	1
BC16	1	0	0	0
BC17	3	1	1	1

### Lactobacilli metabolome correlates with taxonomy and fungistatic/fungicidal activity

We sought a metabolic description of the CFS of the vaginal lactobacilli isolated in the present study. *L*. *gasseri* BC14 was not included in the metabolomics analysis because the metabolic profile of BHI supernatant could not be compared with the metabolic profiles of MRS supernatants.

**Fig 1:**
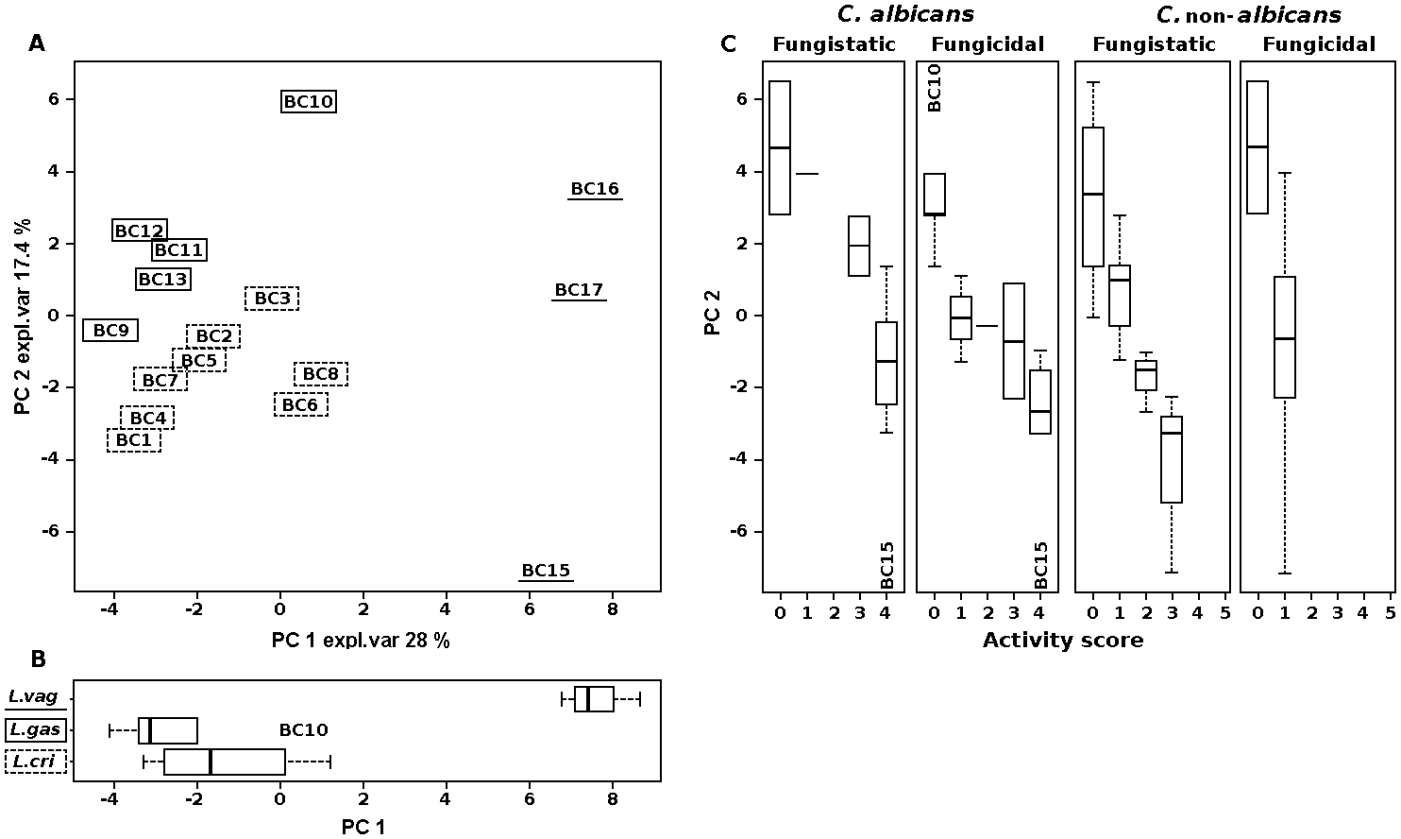
Correlation between metabolome of lactobacilli and fungistatic/fungicidal activity towards *C*. *albicans* and *C*. non-*albicans*. (A) Biplot of a PCA performed on the total metabolites identified by ^1^H-NMR in *Lactobacillus* cell free supernatants. Expl. Var, explained variance. (B) Box plots representing the distribution of *Lactobacillus* species in relation to the metabolome. Lines within the boxes indicate the median values of the samples groups corresponding to *L*. *crispatus*, *L*. *gasseri* and *L*. *vaginalis* species. (C) Box plots representing the distribution of fungistatic/fungicidal activity scores towards *C*. *albicans* and *C*. non-*albicans* in relation to the metabolome. Lines within the boxes indicate the median values of the samples groups corresponding to the different activity scores (0–4 for *C*. *albicans*; 0–5 for *C*. non-*albicans*). Each box represents the interquartile range (25–75th percentile). The bottom and top bars indicate the 10th and 90th percentiles, respectively. Outlier values are indicated (BC10 and BC15).

We identified 40 molecules mainly belonging to the families of aminoacids, organic acids monosaccharides, ketones and alcohols (S3 Table). A Principal Component Analysis (PCA) was performed on entire set of metabolites identified (Fig 1). In the biplot describing the distribution of *Lactobacillus* strains in relation to the pool of metabolites, PC1 and PC2 accounted for the 45.4% of the whole variance of the investigated samples (Fig 1A). This multivariate analysis showed two interesting correlations: (i) metabolome *versus* taxonomy (PC1, expl. var 28%) and (ii) metabolome *versus* fungistatic/fungicidal activity (PC2, expl. var 17.4%). These correlations were best visualized by means of box blots representing the distribution of *Lactobacillus* species (Fig 1B) and fungistatic/fungicidal activity scores (Fig 1C) in relation to the metabolome. Metabolic profiles varied to a greater extent according to the taxonomy. In particular, metabolome of *L*. *vaginalis* significantly differed from those of *L*. *crispatus* and *L*. *gasseri* (*P* < 0.05). The highest metabolic heterogeneity was observed within *L*. *crispatus*, as demonstrated by the width of the corresponding boxplot. Even fungistatic and fungicidal activities of the vaginal lactobacilli were related to their metabolome. Strains with different activity scores were clearly separated in the vertical direction: the most active strains occupied the lower positions while the less active strains were placed in the higher areas of the two-dimensional space represented by the biplot. A linear correlation was observed between median metabolic variance on PC2 and antifungal activity scores against both *C*. *albicans* (fungistatic, *R* ^*2*^ = 0.86, *P* = 0.046; fungicidal, *R* ^*2*^ = 0.83, *P* = 0.02) and *C*. non-*albicans* isolates (fungistatic, *R* ^*2*^ = 0.99, *P* = 0.003). The correlation coefficient related to the fungicidal activity against *C*. non-*albicans* was not calculated due to the presence of only two activity scores (0 and 1).

We searched by Wilcoxon univariate statistical test the metabolites which varied in relation to antifungal activity. We identified 4 metabolites (butyrate, orotate, pyroglutamate, and isoleucine) whose concentrations significantly increased (*P* < 0.05) in CSF of active strains. Fungistatic and fungicidal activities of these compounds were evaluated, but no substance was active at the concentration found in the lactobacilli supernatants. No activity was also observed when a mixture of butyrate, orotate, pyroglutamate and isoleucine was tested, suggesting the lack of synergistic effects. However, we cannot exclude a synergistic action of these metabolites in the more complex cultural medium where other bacterial molecules may act as enhancers.

### Lactobacilli interference with *C*. *albicans* adhesion to HeLa cells

The adhesion of vaginal lactobacilli to epithelial tissue represents the first step in the formation of a barrier to prevent undesirable microbial colonization [22–24].

**Fig 2:**
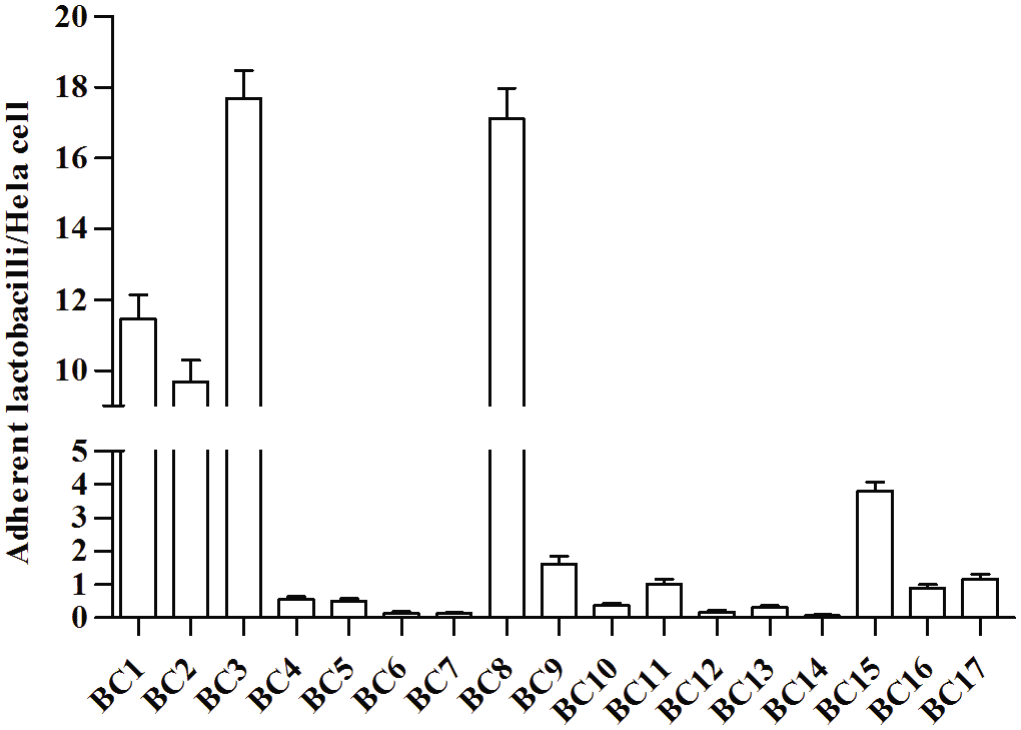
Adhesion of vaginal lactobacilli to HeLa cells. The results were expressed as average number of adherent bacteria per cell. Error bars represent SEM.

Firstly, *Lactobacillus* strains were examined for their ability to adhere to HeLa cells, a cell line that originated from a human carcinoma of the cervix (Fig 2). Adherence varied greatly among the lactobacilli analysed, in a range between 0.07 ± 0.03 and 17.68 ± 0.78 (mean ± SEM) bacteria/cell. *Lactobacillus crispatus* BC1, *L*. *crispatus* BC3 and *L*. *gasseri* BC8 were the most adhesive strains (> 10 bacteria/cell), *L*. *crispatus* BC2 and *L*. *vaginalis* BC15 showed an intermediate adhesiveness (2–10 bacteria/cell), whereas the remaining strains adhered at low levels (< 2 bacteria/cell). These data demonstrate that the adhesive properties are strain-specific rather than species-specific, varying considerably between strains of the same species.

**Fig 3:**
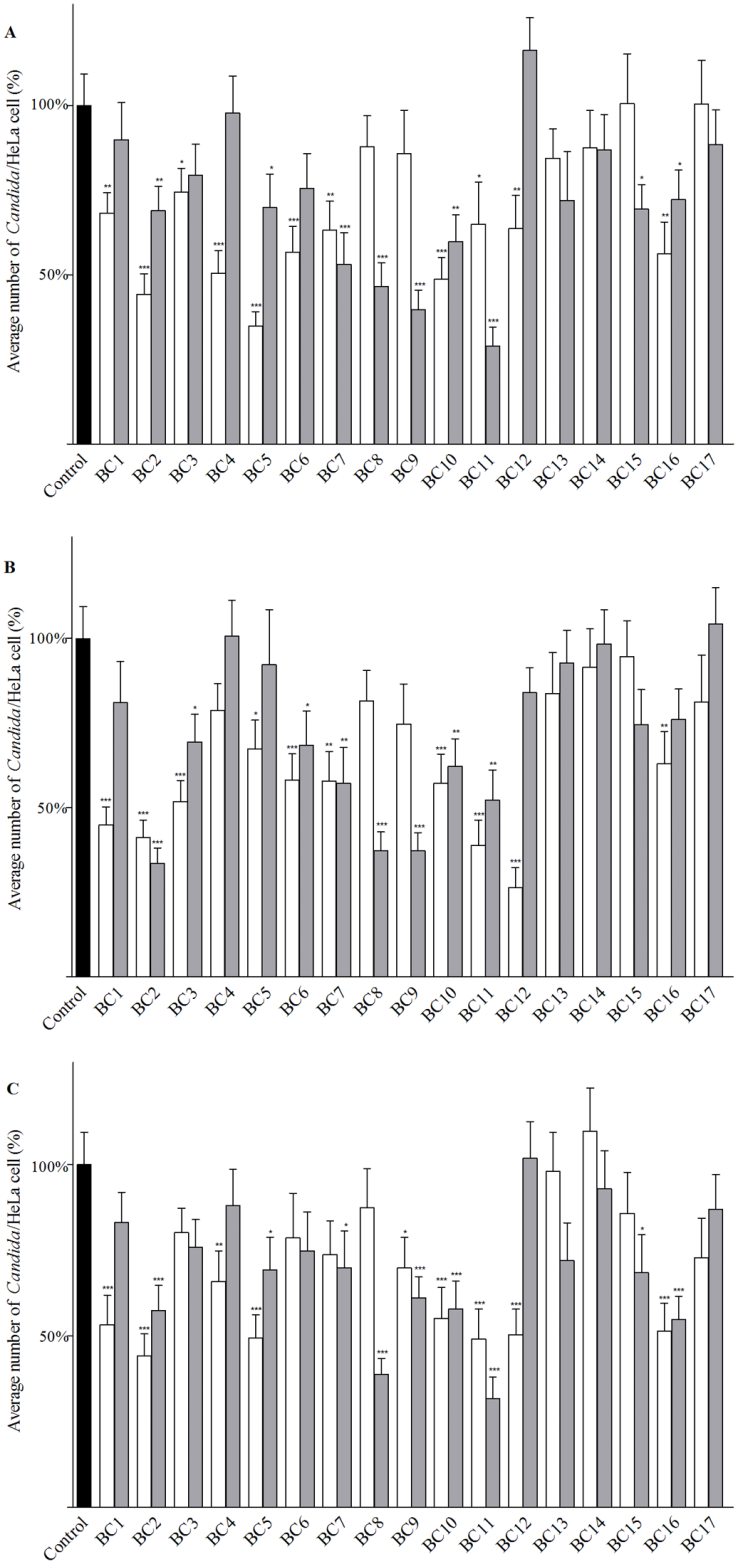
Interference of vaginal lactobacilli with *C*. *albicans* adhesion to HeLa cells. Exclusion (A), competition (B) and displacement (C) experiments were performed. The results were expresses as percentages of adherent yeasts per HeLa cell and compared with adhesion without lactobacilli (control value). The control value was taken as 100% of adhesion (black bars). White and grey bars show the adhesion of *C*. *albicans* in presence of *Lactobacillus* cells and supernatants, respectively. Statistical significance was determined at *P* < 0.05*, *P* < 0.01** and *P* < 0.001***. Error bars represent SEM.

To verify the antagonist effect of the vaginal lactobacilli against *C*. *albicans*, the influence of CP and their respective CFS on the adhesion capacity of the yeast to HeLa cells was investigated (Fig 3). Three mechanisms of inhibition were examined: exclusion (Fig 3A), competition (Fig 3B) and displacement (Fig 3C). Ten strains (BC1, BC2, BC5, BC7, BC8, BC9, BC10, BC11, BC12 and BC16) significantly reduced the adhesion of *C*. *albicans* through all three mechanisms. The inhibitory effect was exerted in some cases by CP and in other cases by CFS. In particular, the interference by *L*. *crispatus* BC2, *L*. *gasseri* BC10 and *L*. *gasseri* BC11 was mediated by both CP and CFS, suggesting that these strains were the most active in terms of inhibition of the pathogen adhesion. Interestingly, BC2, BC10 and BC11 were not the most adhesive strains, as shown by the Fig 2. Only three strains did not exercise any effect on *C*. *albicans* adhesion: *L*. *gasseri* BC13, *L*. *gasseri* BC14 and *L*. *vaginalis* BC17. The remaining strains showed an intermediate behaviour exerting inhibition through one or two mechanisms.

### Lactobacilli effects on *C*. *albicans* histone acetylation

Bothe the yeast histone deacetylase (HDAC) inhibition, and the consequent histone hyper-acetylation, represent a novel mechanism by which *Candida* growth and adhesion to the host epithelium can be reduced [25–27]. Thus, we wondered if the fungistatic/fungicidal activity and interference with *Candida* adhesion exerted by the vaginal lactobacilli could be associated to this mechanism.

Acetylation profiles of H2/H3 and H4 histones of *C*. *albicans* 1 were evaluated for all lactobacilli CFS, except for *L*. *gasseri* BC14 due to the inability of this strain to grow in MRS. We attributed hyper-acetylating ability to strains that determined acetylation levels of H2/H3 or H4 histones at least equal to those induced by sodium butyrate, used as positive control (Table 4). The majority of lactobacilli caused histone hyper-acetylation. In detail, histones H2/H3 were hyper-acetylated by 12 strains and histone H4 by 11 strains. *Lactobacillus gasseri* BC13 and *L*. *vaginalis* BC17 were the only strains that did not cause acetylation of any kind of histone. Notably, these lactobacilli showed no fungicidal activity towards *C*. *albicans* 1 nor any inhibitory effect on the adhesion of the pathogen. These data suggest that the complete lack of inhibition of HDACs could compromise antifungal activity of lactobacilli.

**Table 4:** Acetylation of *C*. *albicans* histones H2/H3 and H4 by lactobacilli cell free supernatants.

**Stimulus**	**Histone acetylation**	
	H2/H3	H4
NaBu	+	+
*S*. *aureus*	-	-
BC1	-	+
BC2	+	-
BC3	+	-
BC4	+	+
BC5	+	-
BC6	+	+
BC7	+	+
BC8	+	+
BC9	+	+
BC10	+	+
BC11	-	+
BC12	+	+
BC13	-	-
BC15	+	+
BC16	+	+
BC17	-	-

NaBu, Sodium Butyrate 20 mM (positive control); +, acetylation ≥ NaBu;-, acetylation < NaBu.

## Discussion

The vaginal mucosa is inhabited by both bacteria and fungi, which normally coexist with the host in a tightly regulated manner. Under certain circumstances this ecological balance may break and turn into a pathological state. A decrease in the number of lactobacilli among the vaginal microbiota may be an aid in the transmission of genitourinary pathogens, including *Candida* spp. While the treatment of VVC by conventional drugs is relatively effective, it has been suggested that women could benefit from restoration of the vaginal communities via supplementation with probiotics [41]. The aim of the present study was to isolate vaginal lactobacilli from healthy women, to characterize them at a molecular level and to evaluate their anti-*Candida* proprieties, in the perspective to develop successful vaginal probiotics for VVC management, considering that the drug-resistances are at present a major problem for the public health systems [17].

In this context, we isolated strains belonging to *L*. *crispatus*, *L*. *gasseri* and *L*. *vaginalis* species. The isolation of *L*. *crispatus* has been strongly associated with a normal vaginal microbiota and absence of vaginal dysbiosis [42]. Longitudinal studies have also shown that the presence of *L*. *crispatus* promotes stability of the vaginal microbiota [43]. Despite the high incidence of *L*. *iners* in the human vaginal microbiota, as detected by culture-independent molecular studies [4, 44], we did not obtain isolates belonging to this species probably because of its stringent nutritional requirements and very low oxygen tolerance [45]. On the other hand, as our goal was to identify health-promoting lactobacilli, *L*. *iners* was of little interest given its close correlation with vaginal dysbiosis [46].

In view of potential application of the isolated *Lactobacillus* strains as vaginal probiotics, we sought to characterize the capacity of these strains to modify the host microenvironment and therefore deliver health benefits. Hydrogen peroxide and lactate are classically associated with the antimicrobial properties of the genus *Lactobacillus* [5]. All strains produced hydrogen peroxide in agreement with the assumption that the vaginal microbiota of healthy women is dominated by H_2_O_2_-producing lactobacilli [35]. Also lactate was produced by all lactobacilli, while butyrate was produced only by certain strains at concentrations that varied significantly depending on the activity score. Since butyrate is a known HDAC inhibitor [29], we hypothesize that it may enhance the anti-*Candida* activity of lactobacilli through the mechanism of histone hyperacetylation.

The fungistatic and fungicidal activities of the vaginal lactobacilli were evaluated against *C*. *albicans* and *C*. non-*albicans*. Compared to previous studies focused on the antifungal activity of lactobacilli [47, 48], our work has the additional value of examining *Lactobacillus* isolates of vaginal source against a broad spectrum of *Candida* species, including the most represented species responsible for gynaecological infections. Therefore, the results obtained in this work provide important information about the real applicability of vaginal lactobacilli in the prevention and treatment of VVC. The broadest spectrum of activity was observed for *L*. *crispatus* BC1, BC4, BC5 and *L*. *vaginalis* BC15, exhibiting fungicidal activity against all isolates of *C*. *albicans* and *C*. *lusitaniae*. Among these strains, *L*. *crispatus* BC1 and *L*. *vaginalis* BC15 exhibited the best anti-*Candida* profile covering also *C*. *tropicalis* and *C*. *glabrata*, albeit in fungistatic mode.

In order to interpret, through a metabolic key, the differences in fungistatic/fungicidal power of the vaginal lactobacilli, we studied by ^1^H-NMR their metabolome and we looked for correlations with taxonomy and activity score. The strong correlation between metabolic profile and taxonomy highlighted the inter-specific variability of bacterial metabolism. Metabolic variance was also related to antifungal activity scores, confirming the excellent antifungal profile of the majority of *L*. *crispatus* strains and *L*. *vaginalis* BC15. These data highlight the potential of metabolomics to measure the taxonomic distance between different *Lactobacillus* strains and predict their anti-*Candida* activity. Although metabolomics has been applied to evaluate the impact of probiotics on the host organism [49], to our knowledge this is the first study employing a metabolomic approach to investigate the antimicrobial activity of health-promoting bacteria, representing a new idea for future researches.

Impairment of pathogens adherence to human cells is considered of major importance for the *in vitro* evaluation of probiotic properties [50]. Most of *Lactobacillus* strains significantly reduced *C*. *albicans* adhesion through every mechanism including exclusion, competition and displacement. In particular, *L*. *crispatus* BC2, *L*. *gasseri* BC10 and *L*. *gasseri* BC11 appeared to be the most active in reducing pathogen adhesion, as their effects were mediated by both cells and supernatants. Interestingly, BC2, BC10 and BC11 were not the most adhesive strains suggesting that the inhibitory effects are not merely due to steric encumbrance and saturation of the adhesion sites, but rather to a reduction of the adherence of the pathogen itself and/or to modifications of the epithelial cells surface. Furthermore, BC2, BC10 and BC11 were not the best performing strains in terms of fungistatic/fungicidal activity. This finding suggest that lactobacilli isolated from healthy vagina can carry out their protective function against *Candida* infection exploiting one particular strategy (inhibition of growth or adhesion) rather than through the combination of two complementary mechanisms.

Inhibition of HDACs can impair fungal growth and adherence to host cells [25–27, 29]. For this reason, we investigated whether the antagonism towards *C*. *albicans* could be associated with histone hyper-acetylation. We observed that histone hyper-acetylation was a widespread prerogative among lactobacilli of vaginal source. Notably, the only strains that did not cause any kind of histone acetylation (*L*. *gasseri* BC13 and *L*. *vaginalis* BC17) were the same ones that did not exercise any inhibitory effect on *Candida* adhesion nor a fungicidal activity, suggesting that inhibition of HDACs could support antifungal activity of vaginal lactobacilli.

Further studies are necessary for a thorough understanding of the antifungal mechanisms of vaginal lactobacilli, i.e. analysis of the antimicrobial activity of the cell-free supernatants to identify specific classes of bioactive molecules, development of a vaginal model with simulated vaginal fluids and *in vivo* tests with animals. However, the findings from this work have enabled us to achieve two important objectives: (i) identify vaginal lactobacilli active against *Candida* spp. and (ii) characterize the mechanisms of action underlying antagonism toward pathogen. The application that follows is the combination of strains exerting different modes of action in order to obtain a probiotic blend with enhanced therapeutic properties. In particular, we have identified strains with a good spectrum of fungistatic/fungicidal activity (*L*. *crispatus* BC1 and *L*. *vaginalis* BC15) that may be associated with strains particularly active in reducing the adhesion of the pathogen (*L*. *crispatus* BC2, *L*. *gasseri* BC10, *L*. *gasseri* BC11). The choice of different species is also an added advantage as it ensures a wider expression of metabolic functions.

## Supporting Information

S1 Table *Candida* isolates used in the present study. Species, strain and origin of the isolates are reported.
(DOCX)

S2 Table Fungistatic activity of cell free supernatants of *Lactobacillus* strains towards *Candida* isolates. Turbidity values of *Candida* cultures exposed to cell free supernatants of lactobacilli for 24 h are reported. *Candida* cultures grown in MRS broth were used as control (No lactobacilli). Data are expressed as OD_450nm_ median values ± Standard Deviation. Experiments were performed at least in triplicate.
(DOCX)

S3 Table Metabolites identified by ^1^H-NMR in cell free supernatants of vaginal lactobacilli. Concentrations were calculated as differences from MRS medium. Values are expressed as mmol/l.
(DOCX)
