# Supplementary material for: Isolation of Vaginal Lactobacilli and Characterization of Anti-Candida Activity
Source: PLoS One. 2015 Jun 22;10(6):e0131220. doi: 10.1371/journal.pone.0131220 (PMC4476673; doi:10.1371/journal.pone.0131220)
Supplement: S1 Table — Species, strain and origin of the isolates are reported. (DOCX) [file pone.0131220.s001.docx]

**S1 Table. *Candida* isolates used in the present study.** Species, strain and origin of the isolates are reported.

| Species | Strain | Origin |
| --- | --- | --- |
| *C. albicans* | 001-14 | Vaginal swab |
| *C. albicans* | 002-14 | Vaginal swab |
| *C. albicans* | 003-14 | Vaginal swab |
| *C. albicans* | 004-14 | Vaginal swab |
| *C. tropicalis* | 005-14 | Vaginal swab |
| *C. krusei* | 006-14 | Vaginal swab |
| *C. parapsilosis* | 007-14 | Vaginal swab |
| *C. glabrata* | 008-14 | Vaginal swab |
| *C. lusitaniae* | 009-14 | Vaginal swab |
