# Supplementary material for: Isolation of Vaginal Lactobacilli and Characterization of Anti-Candida Activity
Source: PLoS One. 2015 Jun 22;10(6):e0131220. doi: 10.1371/journal.pone.0131220 (PMC4476673; doi:10.1371/journal.pone.0131220)
Supplement: S2 Table — Turbidity values of Candida cultures exposed to cell free supernatants of lactobacilli for 24 h are reported. Candida cultures grown in MRS broth were used as control (No lactobacilli). Data are expressed as OD450nm median values ± Standard Deviation. Experiments were performed at least in triplicate. (DOCX) [file pone.0131220.s002.docx]

**S2 Table.** **Fungistatic activity of cell free supernatants of *Lactobacillus* strains towards *Candida* isolates.** Turbidity values of *Candida* cultures exposed to cell free supernatants of lactobacilli for 24 h are reported. *Candida* cultures grown in MRS broth were used as control (No lactobacilli). Data are expressed as OD_450nm_ median values ± Standard Deviation. Experiments were performed at least in triplicate.

|  | *Candida* isolate | | | | | | | | |
| --- | --- | --- | --- | --- | --- | --- | --- | --- | --- |
| *Lactobacillus* strain | *C. albicans 1* | *C. albicans 2* | *C. albicans 3* | *C. albicans 4* | *C. tropicalis* | *C. krusei* | *C. parapsilosis* | *C. glabrata* | *C. lusitaniae* |
| No lactobacilli | 0.837 ± 0.048 | 0.743 ± 0.058 | 0.789 ± 0.087 | 0.787 ± 0.050 | 0.712 ± 0.078 | 0.709 ± 0.094 | 0.518 ± 0.084 | 0.905 ± 0.093 | 0.613 ± 0.078 |
| BC1 | 0.262 ± 0.030 | 0.263 ± 0.038 | 0.249 ± 0.045 | 0.258 ± 0.049 | 0.322 ± 0.07 | 0.371 ± 0.069 | 0.303 ± 0.038 | 0.344 ± 0.053 | 0.297 ± 0.05 |
| BC2 | 0.291 ± 0.076 | 0.302 ± 0.064 | 0.27 ± 0.068 | 0.26 ± 0.065 | 0.444 ± 0.047 | 0.521 ± 0.063 | 0.38 ± 0.07 | 0.564 ± 0.075 | 0.294 ± 0.06 |
| BC3 | 0.241 ± 0.024 | 0.229 ± 0.013 | 0.337 ± 0.110 | 0.284 ± 0.082 | 0.374 ± 0.036 | 0.487 ± 0.129 | 0.333 ± 0.019 | 0.482 ± 0.124 | 0.242 ± 0.01 |
| BC4 | 0.295 ± 0.070 | 0.261 ± 0.064 | 0.283 ± 0.085 | 0.274 ± 0.069 | 0.368 ± 0.043 | 0.449 ± 0.078 | 0.404 ± 0.075 | 0.438 ± 0.076 | 0.306 ± 0.066 |
| BC5 | 0.264 ± 0.038 | 0.277 ± 0.061 | 0.263 ± 0.057 | 0.274 ± 0.063 | 0.34 ± 0.051 | 0.442 ± 0.04 | 0.37 ± 0.052 | 0.458 ± 0.015 | 0.306 ± 0.063 |
| BC6 | 0.274 ± 0.022 | 0.259 ± 0.014 | 0.341 ± 0.070 | 0.311 ± 0.082 | 0.35 ± 0.03 | 0.428 ± 0.082 | 0.324 ± 0.066 | 0.452 ± 0.081 | 0.262 ± 0.023 |
| BC7 | 0.272 ± 0.034 | 0.293 ± 0.069 | 0.283 ± 0.041 | 0.283 ± 0.046 | 0.329 ± 0.009 | 0.4 ± 0.052 | 0.364 ± 0.052 | 0.418 ± 0.03 | 0.308 ± 0.057 |
| BC8 | 0.246 ± 0.009 | 0.248 ± 0.008 | 0.284 ± 0.046 | 0.255 ± 0.015 | 0.382 ± 0.059 | 0.496 ± 0.095 | 0.33 ± 0.039 | 0.527 ± 0.122 | 0.26 ± 0.007 |
| BC9 | 0.386 ± 0.076 | 0.306 ± 0.066 | 0.367 ± 0.099 | 0.365 ± 0.097 | 0.426 ± 0.059 | 0.477 ± 0.086 | 0.442 ± 0.055 | 0.546 ± 0.116 | 0.307 ± 0.055 |
| BC10 | 0.588 ± 0.072 | 0.555 ± 0.026 | 0.572 ± 0.023 | 0.612 ± 0.007 | 0.593 ± 0.033 | 0.671 ± 0.057 | 0.544 ± 0.086 | 0.795 ± 0.07 | 0.506 ± 0.01 |
| BC11 | 0.518 ± 0.030 | 0.456 ± 0.080 | 0.528 ± 0.018 | 0.561 ± 0.029 | 0.504 ± 0.046 | 0.63 ± 0.059 | 0.415 ± 0.066 | 0.661 ± 0.107 | 0.329 ± 0.081 |
| BC12 | 0.418 ± 0.028 | 0.387 ± 0.111 | 0.383 ± 0.064 | 0.314 ± 0.075 | 0.499 ± 0.074 | 0.555 ± 0.085 | 0.387 ± 0.049 | 0.471 ± 0.076 | 0.296 ± 0.061 |
| BC13 | 0.303 ± 0.054 | 0.287 ± 0.052 | 0.236 ± 0.038 | 0.283 ± 0.056 | 0.431 ± 0.061 | 0.417 ± 0.005 | 0.39 ± 0.084 | 0.612 ± 0.082 | 0.303 ± 0.056 |
| BC14 | 0.403 ± 0.045 | 0.446 ± 0.055 | 0.427 ± 0.058 | 0.431 ± 0.060 | 1.301 ± 0.149 | 1.356 ± 0.227 | 0.41 ± 0.031 | 0.44 ± 0.068 | 0.805 ± 0.083 |
| BC15 | 0.238 ± 0.011 | 0.241 ± 0.013 | 0.246 ± 0.013 | 0.252 ± 0.013 | 0.356 ± 0.101 | 0.439 ± 0.097 | 0.32 ± 0.04 | 0.383 ± 0.061 | 0.249 ± 0.012 |
| BC16 | 0.417 ± 0.053 | 0.453 ± 0.07 | 0.422 ± 0.057 | 0.429 ± 0.072 | 0.455 ± 0.081 | 0.468 ± 0.08 | 0.43 ± 0.017 | 0.523 ± 0.082 | 0.35 ± 0.071 |
| BC17 | 0.372 ± 0.045 | 0.32 ± 0.076 | 0.414 ± 0.002 | 0.358 ± 0.086 | 0.398 ± 0.034 | 0.5 ± 0.091 | 0.365 ± 0.01 | 0.508 ± 0.087 | 0.287 ± 0.07 |
