# Supplementary material for: Isolation of Vaginal Lactobacilli and Characterization of Anti-Candida Activity
Source: PLoS One. 2015 Jun 22;10(6):e0131220. doi: 10.1371/journal.pone.0131220 (PMC4476673; doi:10.1371/journal.pone.0131220)
Supplement: S3 Table — Concentrations were calculated as differences from MRS medium. Values are expressed as mmol/l. (DOCX) [file pone.0131220.s003.docx]

**S3 Table. Metabolites identified by ^1^H-NMR in cell free supernatants of vaginal lactobacilli.** Concentrations were calculated as differences from MRS medium. Values are expressed as mmol/l.

| **Strain** | **Molecule** | | | | | | | |
| --- | --- | --- | --- | --- | --- | --- | --- | --- |
|  | **2-Hydroxybutyrate** | **Leucine** | **Isoleucine** | **Valine** | **Ethanol** | **Propylene glycol** | **Alanine** | **Valerate** |
| **BC1** | -0.660 | 0.859 | 0.409 | 0.549 | 0.079 | 0.007 | 1.011 | -0.088 |
| **BC2** | -0.367 | 1.204 | 0.381 | 0.509 | 0.052 | 0.019 | 0.995 | -0.070 |
| **BC3** | 1.525 | 3.435 | 1.102 | 0.890 | 0.474 | -0.007 | 0.829 | -0.204 |
| **BC4** | -0.718 | 0.568 | 0.231 | 0.351 | 0.152 | 0.004 | 0.611 | 0.095 |
| **BC5** | -0.266 | 1.283 | 0.244 | 0.334 | 0.004 | 0.013 | 0.646 | 0.116 |
| **BC6** | 0.153 | 0.942 | 0.225 | 0.258 | -0.019 | 0.034 | 0.027 | 0.417 |
| **BC7** | 1.314 | 3.395 | 0.737 | 0.628 | 0.494 | 0.004 | 1.000 | -0.202 |
| **BC8** | 2.095 | 3.354 | 0.908 | 0.838 | 0.206 | 0.030 | 0.402 | 0.482 |
| **BC9** | -0.916 | 0.663 | 0.344 | 0.550 | 0.510 | 0.011 | 1.304 | -0.266 |
| **BC10** | -0.781 | 0.742 | 0.519 | 0.571 | 0.126 | 0.024 | 0.904 | 0.044 |
| **BC11** | -0.481 | 1.342 | 0.632 | 0.691 | 0.009 | 0.016 | 0.860 | 0.057 |
| **BC12** | -0.456 | 1.346 | 0.770 | 0.825 | 0.071 | 0.009 | 1.019 | 0.130 |
| **BC13** | -0.466 | 1.478 | 0.584 | 0.892 | 0.158 | 0.006 | 1.561 | 0.175 |
| **BC15** | 0.962 | 0.496 | 0.432 | 0.068 | 10.098 | 0.138 | -0.182 | 0.194 |
| **BC16** | 1.665 | 1.962 | 0.827 | 0.687 | 36.180 | 0.325 | 1.325 | 0.068 |
| **BC17** | 1.999 | 2.925 | 0.950 | 0.761 | 43.735 | 0.218 | 0.311 | 0.456 |

| **Strain** | **Molecule** | | | | | | | |
| --- | --- | --- | --- | --- | --- | --- | --- | --- |
|  | **Acetate** | **Methionine** | **Acetoin** | **Acetone** | **Pyruvate** | **Sarcosine** | **Aspartate** | **N.N-dimethylglycine** |
| **BC1** | -29.207 | 0.119 | 0.028 | 0.644 | 0.900 | 0.150 | -0.022 | 0.000 |
| **BC2** | -23.143 | 0.157 | 0.021 | 0.464 | 0.704 | 0.148 | -0.012 | -0.001 |
| **BC3** | -17.856 | 0.218 | 0.123 | 0.026 | 0.767 | 0.111 | -0.010 | 0.093 |
| **BC4** | -27.973 | 0.198 | 0.013 | 0.415 | 0.166 | 0.100 | -0.030 | 0.001 |
| **BC5** | -23.890 | 0.200 | 0.018 | 0.334 | 0.332 | 0.101 | -0.025 | 0.002 |
| **BC6** | -26.057 | 0.158 | -0.009 | 0.007 | 0.305 | 0.115 | -0.018 | -0.005 |
| **BC7** | -32.954 | 0.068 | 0.092 | 1.798 | 0.151 | 0.077 | -0.025 | 0.003 |
| **BC8** | -30.984 | 0.243 | 0.016 | -0.004 | 0.124 | 0.142 | -0.029 | -0.002 |
| **BC9** | -36.668 | 0.196 | 1.982 | 2.952 | 0.173 | 0.161 | -0.022 | -0.001 |
| **BC10** | -22.914 | 0.256 | 0.003 | 0.363 | 0.248 | 0.126 | -0.001 | 0.001 |
| **BC11** | -24.802 | 0.274 | 0.020 | 0.410 | 0.403 | 0.135 | 0.005 | 0.001 |
| **BC12** | -32.121 | 0.281 | 0.061 | 0.837 | 0.415 | 0.139 | -0.021 | 0.000 |
| **BC13** | -30.403 | 0.316 | 0.010 | 0.526 | 0.194 | 0.138 | -0.026 | 0.116 |
| **BC15** | -5.218 | -0.137 | -0.004 | -0.057 | 0.294 | 0.019 | 0.018 | 0.035 |
| **BC16** | -11.551 | -0.061 | -0.012 | 0.006 | 0.245 | 0.028 | 0.025 | 0.077 |
| **BC17** | -13.631 | 0.066 | -0.009 | -0.008 | 0.140 | 0.081 | -0.001 | -0.004 |

| **Strain** | **Molecule** | | | | | | | |
| --- | --- | --- | --- | --- | --- | --- | --- | --- |
|  | **Creatine** | **Creatinine** | **Choline** | **sn-glycero-3-phosphocholine** | **Fructose** | **Pyroglutamate** | **1,3-dihydroxyacetone** | **Lactose~~4.45~~** |
| **BC1** | -0.190 | -0.296 | 0.011 | -0.196 | -0.127 | -0.158 | 1.105 | +0.598 |
| **BC2** | -0.247 | -0.293 | 0.017 | -0.183 | -0.072 | 0.246 | 0.792 | 0.726 |
| **BC3** | -0.352 | -0.481 | -0.011 | -0.196 | -0.006 | -0.489 | 0.962 | 0.340 |
| **BC4** | -0.295 | -0.332 | 0.038 | -0.222 | -0.015 | -0.195 | 0.789 | 0.792 |
| **BC5** | -0.339 | -0.366 | 0.035 | -0.152 | -0.108 | -0.431 | 0.761 | 0.756 |
| **BC6** | -0.359 | -0.415 | -0.008 | -0.177 | 0.109 | -0.625 | 0.561 | 0.266 |
| **BC7** | -0.308 | -0.462 | 0.039 | -0.171 | 0.127 | -0.171 | 0.794 | 0.604 |
| **BC8** | -0.328 | -0.395 | -0.009 | -0.217 | -0.022 | -0.710 | 0.908 | -2.110 |
| **BC9** | -0.164 | -0.353 | 0.029 | -0.230 | 0.125 | 1.280 | 1.279 | 1.198 |
| **BC10** | 0.302 | -0.084 | 0.003 | -0.140 | -0.018 | -0.268 | 0.916 | 0.437 |
| **BC11** | -0.358 | -0.360 | 0.007 | -0.212 | 0.043 | -0.232 | 1.392 | 0.519 |
| **BC12** | -0.340 | -0.343 | 0.007 | -0.241 | -0.021 | -0.714 | 1.192 | 0.500 |
| **BC13** | -0.271 | -0.310 | 0.051 | -0.204 | -0.031 | -0.815 | 1.916 | 1.155 |
| **BC15** | -0.229 | -0.372 | -0.155 | -0.178 | -0.068 | -0.604 | 0.372 | -1.087 |
| **BC16** | 0.133 | -0.031 | -0.071 | -0.211 | -0.143 | 0.054 | 0.136 | -0.303 |
| **BC17** | 0.109 | -0.122 | -0.022 | -0.146 | -0.120 | 0.106 | 0.388 | -2.079 |

| **Strain** | **Molecule** | | | | | | | |
| --- | --- | --- | --- | --- | --- | --- | --- | --- |
|  | **Glucose** | **Ribose** | **Uracil** | **2-deoxyuridine** | **Uridine** | **Adenosine** | **Inosine** | **Orotate** |
| **BC1** | -28.537 | 0.144 | -0.093 | -0.072 | -0.012 | 0.146 | -0.110 | 0.029 |
| **BC2** | -20.886 | 0.118 | -0.062 | -0.062 | -0.017 | 0.108 | -0.092 | 0.030 |
| **BC3** | -18.982 | 0.111 | -0.053 | -0.062 | -0.011 | 0.074 | -0.063 | 0.007 |
| **BC4** | -24.024 | 0.082 | -0.055 | -0.049 | -0.013 | 0.083 | -0.086 | 0.029 |
| **BC5** | -19.468 | 0.056 | -0.048 | -0.049 | -0.007 | 0.053 | -0.079 | 0.017 |
| **BC6** | -19.164 | 0.053 | -0.068 | -0.043 | 0.008 | 0.045 | -0.061 | 0.022 |
| **BC7** | -23.638 | 0.088 | -0.044 | -0.056 | -0.012 | 0.058 | -0.092 | 0.016 |
| **BC8** | -19.655 | 0.064 | -0.085 | -0.054 | -0.011 | 0.050 | -0.063 | 0.022 |
| **BC9** | -26.730 | 0.003 | -0.063 | -0.031 | -0.032 | 0.037 | -0.038 | 0.007 |
| **BC10** | -6.108 | 0.039 | 0.045 | -0.039 | -0.020 | 0.014 | -0.051 | 0.005 |
| **BC11** | -18.104 | 0.036 | -0.012 | -0.059 | -0.027 | 0.032 | -0.052 | 0.003 |
| **BC12** | -21.396 | 0.061 | 0.040 | -0.075 | -0.019 | 0.061 | -0.023 | 0.001 |
| **BC13** | -28.827 | 0.011 | -0.097 | -0.040 | -0.039 | -0.013 | -0.010 | 0.008 |
| **BC15** | -26.413 | 0.032 | -0.092 | 0.030 | -0.025 | 0.004 | -0.087 | 0.027 |
| **BC16** | -16.413 | 0.046 | -0.080 | -0.010 | -0.014 | -0.006 | -0.038 | 0.003 |
| **BC17** | -20.805 | 0.031 | -0.086 | -0.024 | -0.017 | -0.019 | -0.072 | 0.009 |

| **Strain** | **Molecule** | | | | | | | |
| --- | --- | --- | --- | --- | --- | --- | --- | --- |
|  | **N-Acetyltyrosine** | **Tyrosine** | **Tryptophan** | **Phenylalanine** | **Cytidine** | **Formate** | **Lactate** | **Butyrate** |
| **BC1** | -0.468 | 0.026 | -0.147 | -0.429 | -0.057 | -0.158 | 2.910 | 0.348 |
| **BC2** | -0.048 | 0.413 | -0.172 | -0.038 | -0.056 | -0.166 | 6.830 | 0.000 |
| **BC3** | -0.680 | 0.396 | -0.652 | 0.129 | -0.055 | -0.086 | 9.450 | 0.000 |
| **BC4** | -0.111 | 0.469 | -0.024 | 0.172 | -0.072 | -0.173 | 3.320 | 0.354 |
| **BC5** | -0.543 | 0.112 | -0.068 | 0.157 | -0.059 | -0.159 | 5.100 | 0.125 |
| **BC6** | -0.065 | 0.132 | -0.196 | 0.126 | -0.051 | -0.161 | 7.870 | 0.833 |
| **BC7** | -0.041 | 0.244 | -0.118 | -0.041 | -0.069 | -0.172 | 1.420 | 0.014 |
| **BC8** | -0.125 | 0.234 | -0.300 | 0.033 | -0.052 | -0.165 | 3.050 | 0.464 |
| **BC9** | -0.406 | 0.026 | -0.181 | -0.294 | -0.058 | -0.135 | 4.750 | 0.000 |
| **BC10** | -0.169 | 0.627 | -0.209 | 0.745 | -0.039 | -0.192 | 9.400 | 0.000 |
| **BC11** | -0.211 | 0.618 | -0.245 | 0.641 | -0.059 | -0.162 | 14.600 | 0.000 |
| **BC12** | -0.146 | 0.531 | -0.238 | 0.521 | -0.065 | -0.163 | 9.470 | 0.000 |
| **BC13** | -0.592 | 0.701 | -0.101 | -0.178 | -0.069 | -0.132 | 1.620 | 0.0184 |
| **BC15** | 0.081 | -0.221 | -0.092 | -1.304 | -0.053 | -0.059 | 47.400 | 0.642 |
| **BC16** | -0.149 | 0.453 | -0.596 | -0.095 | -0.026 | 0.147 | 24.400 | 0.000 |
| **BC17** | -0.568 | 0.096 | -0.283 | 0.180 | -0.028 | -0.093 | 23.400 | 0.327 |
